# Correction: Rational design of a “dual lock-and-key” supramolecular photosensitizer based on aromatic nucleophilic substitution for specific and enhanced photodynamic therapy

**DOI:** 10.1039/d0sc90269a

**Published:** 2020-12-08

**Authors:** Kun-Xu Teng, Li-Ya Niu, Yan-Fei Kang, Qing-Zheng Yang

**Affiliations:** Key Laboratory of Radiopharmaceuticals, Ministry of Education, College of Chemistry, Beijing Normal University Beijing 100875 P. R. China niuly@bnu.edu.cn qzyang@bnu.edu.cn

## Abstract

Correction for ‘Rational design of a “dual lock-and-key” supramolecular photosensitizer based on aromatic nucleophilic substitution for specific and enhanced photodynamic therapy’ by Kun-Xu Teng *et al.*, *Chem. Sci.*, 2020, **11**, 9703–9711, DOI: 10.1039/D0SC01122C.

The authors regret an error in Fig. 1f. The correct image is shown below.

**Fig. 1:**
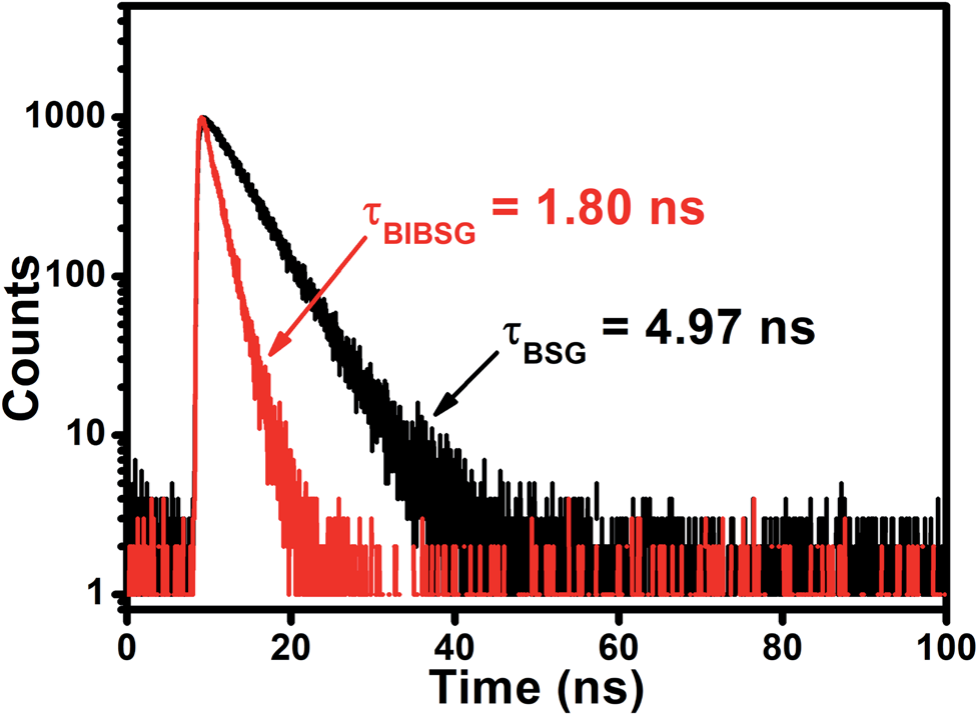
(f) Fluorescence decay curves of BSG and BIBSG, and the detection wavelength is 600 nm in DMSO.

Additionally, there was a minor error in Fig. 4a. The correct image is shown below.

**Fig. 4:**
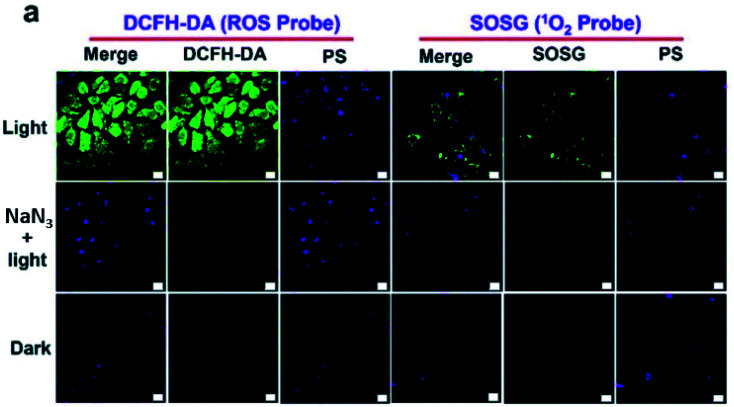
(a) Evaluation of ^1^O_2_ generation in HepG2 cells with DCFH-DA and SOSG. The scale bar represents 20 μm.

The Royal Society of Chemistry apologises for these errors and any consequent inconvenience to authors and readers.

## Supplementary Material

